# Variability of the Chemical Composition of the Essential Oil from the Amazonian Ishpingo Species (*Ocotea quixos*)

**DOI:** 10.3390/molecules26133961

**Published:** 2021-06-28

**Authors:** Eduardo Valarezo, Antoine Vullien, Dayra Conde-Rojas

**Affiliations:** Departamento de Química, Universidad Técnica Particular de Loja, Loja 110150, Ecuador; antoine.vullien@yahoo.fr (A.V.); dayramireya@gmail.com (D.C.-R.)

**Keywords:** essential oil, chemical composition, variability, environmental conditions, *Ocotea quixos*

## Abstract

*Ocotea quixos* (Lam.) Kosterm. is an aromatic tree native to Ecuador, whose leaves are used to prepare aromatic beverages to which different health benefits are attributed. In this study, *Ocotea quixos* leaves were collected in the Amazon region in different environmental conditions and subjected to hydrodistillation to isolate the essential oil. The collection variables used were type of soil, amount of shade, and height; in addition, the presence of twig and leaf age and moisture were used as variables. Chemical composition was analyzed by means of gas chromatography equipped with a flame ionization detector and gas chromatography coupled to mass spectrometry. A wide variety of chemical compositions were detected in the samples. In total, forty-seven compounds were identified, which represented between 97.17% and 99.89% of the total composition. The constituents were mainly grouped into aliphatic sesquiterpene hydrocarbons (33.03–55.89%), other compounds (8.94–47.83%), and oxygenated monoterpenes (1.97–39.66%). The main constituents were found to be (E)-cinnamyl acetate (5.96–41.65%), (E)-methyl cinnamate (0.38–37.91%), and trans-caryophyllene (8.77–37.02%). The statistical analysis suggested the existence of two essential oil chemotypes and a direct correlation between environmental conditions and chemical composition of the essential oils.

## 1. Introduction

Lauraceae is a family of flowering plants belonging to order Laurales; this family comprises approximately 2978 accepted species in nearly 68 genera worldwide [1]. The Lauraceae mostly consist of trees or shrubs, except the species of genus Cassytha (19 species), which are vines or parasitic vines. Species in this family include food plants such as the avocado (*Persea americana*), in addition to several timber trees, spice, and flavoring plants. Due to the presence of aromatic oil glands, these species produce essential oils [2]. The species of the Lauraceae family are distributed from tropical to warm temperate regions, especially Southeast Asia and tropical America [3]. In Ecuador (South America), the Lauraceae family is represented by 15 genera and more than 167 species, of which 23 are endemic and are distributed in all the humid forests of the three natural regions (Costa, Sierra, and Oriente or Amazonia) of continental Ecuador. Some endemic species are part of the humid inter-Andean vegetation [4]. Within the Lauraceae family, the *Ocotea* genus is one of the most abundant in number of species with 428 individuals; these include trees and shrubs and are mostly distributed in tropical and subtropical regions of Central and South America and the West Indies, with some species in Africa, Madagascar, and one in the Macaronesian islands [3]. In Ecuador, the *Ocotea* genus is represented by 40 recognized species [5].

*Ocotea quixos* (Lam.) Kosterm. (class: Equisetopsida C. Agardh; subclass: Magnoliidae Novák ex Takht.; superorder: Magnolianae Takht.; order: Laurales Juss. ex Bercht. and J. Presl; family: Lauraceae Juss.; genus: *Ocotea* Aubl.) is an Ecuadorian-native and cultivated aromatic species, widely distributed in the Andean and Amazonian regions between 0–1000 m a.s.l., especially in the Amazonian provinces of Napo and Sucumbíos [6]. The species is commonly known as “*ishpingo*” (from Quechua ishpinku), “*canela*”, “*canelo*”, “*canelón*”, “*flor de canela*”, and “*canela de la amazonía*” (Spanish language), *ishpinku* or “*ishpingu*” (Kichwa language, dialect of the Amazon region), “*anís ahwa*” (Spanish-Kichwa language), “*ishpink*” (Achuar language), “*cañi*” (Shiwiar language), ishpingk (Shuar language), and “*ocatoe*” (Wao Tededo language). Most of the common names are related to its smell, similar to cinnamon (“*canela*” in Spanish). The *Ocotea quixos* plant is a tree that can measure up to 25 m in height and 80 cm in diameter; its flowers are small and greenish-white in color, and its leaves are up to 15 cm long with a dark-green beam. The fruits are approximately 5 cm long and yellowish-green in color. The leaves and fruit calices from ishpingo are used to prepare aromatic waters. In Ecuador, this species is part of the preparation of ritual foods, such as the traditional drink of the day of the dead, “colada morada”. Ishpingo is also used in some traditional remedies; the infusion is consumed to treat diabetes and to calm body aches, and it is also taken by women after childbirth to improve digestion [7]. Currently, there are no reports regarding toxicity in this species.

Associated with aromatic plants is the presence of essential oils (EOs). Essential oils, also called volatile secondary metabolites, are complex mixtures of compounds between 10 and 25 carbons. The biological properties of EOs are directly related to the chemical nature of the compounds that comprise them. The existence of essential oil in ishpingo leaves has been previously reported [8]. Some important biological properties are attributed to the *O. quixos* essential oil, such as antioxidant activity [9], antifungal activity [10], larvicidal activity [11], antiviral activity [12], and phytotoxicity [13]. The presence of secondary metabolites in plant species changes due to variations in the intrinsic and extrinsic factors, which modifies the biological properties of Eos. For this reason, the objectives of this research were as follows: i. to contribute to the knowledge of the aromatic species of the Lauraceae family by isolating and studying the essential oil of the *Ocotea quixos* from Ecuador, and ii. to determine the influence of environmental conditions on the chemical composition of the essential oil from *Ocotea quixos* leaves.

## 2. Results

Twenty-one samples of ishpingo leaves were analyzed. The samples were collected in different soil types at different shade levels and at different heights; in addition, the influence of leaf age and presence of twigs and moisture were analyzed. Table 1 shows the levels of the six variables used for each sample. From the soil type and shade variables, five levels were used: three levels for the height variable and two levels for the leaf age, branch presence, and moisture variables.

The qualitative and quantitative variability of the essential oil from *O. quixos* leaves is shown in Table 2. In total, forty-seven compounds were identified in ishpingo essential oil, of which fourteen were present in all samples. The chemical nature, chemical formula and monoisotopic mass of the compounds present in ishpingo oil are shown in Table 2. The compounds identified represented between 97.17% and 99.89% of the total composition. The compounds identified in all samples represented between 64.16% and 90.96%. The constituents were mainly grouped into aliphatic sesquiterpene hydrocarbons (ALS, 33.03–55.89%), other compounds (OTC, 8.94–47.83%), and oxygenated monoterpenes (OXM, 1.97–39.66%). Furthermore, low amounts of aromatic monoterpene hydrocarbons (ARM, 0–0.26%) and aromatic sesquiterpene hydrocarbons (ARS, 0–7.48%) were identified in some samples. The main constituents were found to be (E)-cinnamyl acetate (5.96–41.65%, OTC), (E)-methyl cinnamate (0.38–37.91%, OXM), and trans-caryophyllene (8.77–37.02%, ALS).

The five levels of the soil type variable used were as follows: poor clay (Q1) with approximately 30% sand, 25% silt, and 45% clay; clay (Q2) with 25-15-60 sand-silt-clay percentages; rich clay (Q3) (10-10-80); sand (Q4) (85-10-5) and loam (Q5) (40-40-20). For the study on the influence exerted by the type of soil variable on the chemical composition, the ishpingo leaves were collected at a height of 600 ± 30 m a.s.l. under conditions of 70% shade (Table 1).

The main compounds in the essential oils from samples Q1–Q5 collected in the five types of soils were (E)-methyl cinnamate (compound number 15) and trans-Caryophyllene (compound 18) with different concentrations. In Q1 (poor clay), Q2 (clay), and Q4 (sand), the main compound was (E)-methyl cinnamate (compound 15) with 29.79%, 16.00%, and 37.25%, respectively (Table 2). In Q3 (rich clay) with 21.19% and Q5 (loam) soils with 13.41%, the majority compound was trans-caryophyllene (18). According to the soil type, the compounds were mainly grouped into oxygenated monoterpenes (OXM) and aliphatic sesquiterpene hydrocarbons (ALS); the presence of aromatic monoterpene hydrocarbons (ARM) was not detected. A large amount of ALS compounds were identified in the Q2 (54.61%) and Q5 (55.89%) samples.

The variation of the chemical composition of the oil extracted from samples collected at different heights is shown in Table 2. Sampling and extraction of the essential oil were carried out under two conditions. For condition one, the leaves of trees that grew in loam soil with 0% shade (Q6, Q7 and Q8) were collected and, for condition two, those in rich clay soil with 30% shade (Q9 and Q10). 

In essential oils from tree samples growing at heights of 400 (Q6) and 500 m a.s.l. (Q7) in loam soil, the main compound was (E)-cinnamyl acetate (21), followed by trans-caryophyllene (18); at 600 m a.s.l. (Q8) in the same soil type, it was determined that the main compound was compound 18 (17.55%) (Table 2). In rich clay soil with 30% shade at a height of 400 m (Q9), the main compound was determined to be (E)-methyl cinnamate (37.91%), while at 600 m (Q10) it was (E)-cinnamyl acetate (23.53%). In condition one, the chemical compounds were grouped mainly in ALS with percentages of 53.22%, 45.26%, and 50.48% for samples Q6, Q7, and Q8, respectively. In condition two at 400 m (Q9), the compounds were of the oxygenated monoterpenes (OXM, 39.66%) nature and, at 600 m (Q10), the compounds were mainly grouped in aliphatic sesquiterpene hydrocarbons (ALS, 38.78%) and other compounds (OTC, 37.49%) without a significant difference between the two groups.

The study of the influence of the amount of shade on the chemical composition of the oil was carried out using five levels (0, 10, 30, 50 and 70% of shade); the results obtained are shown in Table 2. For analysis, the samples were collected at 600 ± 30 m a.s.l., in loam soil. Under these conditions, the main compound was trans-caryophyllene at 0% of shade (Q11) with 17.55% and at 10% of shade (Q12), with 30.07%. (E)-cinnamyl acetate was the main compound in the 30% shade (Q13) (41.65%) and 50% shade (Q14) (28.62%) samples. In 70% shade (Q15) conditions, the main compound was trans-caryophyllene, but only with 13.41%. The groups of compounds with the highest percentage were ALS due to the contribution of compounds 18 and 25, and other compounds (OTC) for compounds 21 and 28.

The influence of leaf age on the chemical composition is shown in the continuation of Table 2. For this analysis, the samples were collected at 600 ± 30 m a.s.l. in loam soil with 10% shade. In the young (Q16) and old (Q17) leaves, the main compound was trans-caryophyllene, although with the highest percentage in sample Q16. In both cases, the compounds were grouped in ALS with the main contribution of compounds 18 and 25.

Due to the nature of the collection, part of the plant materials collected were branches (twigs) attached to the leaves through the petiole; the difference in chemical composition between the distilled leaves with branches (Q19) and without branches (Q18) is shown in the continuation of Table 2. In addition, this table shows the chemical composition of the samples with two humidity levels: fresh (65 ± 5%) (Q20) and dried (12 ± 2%) (Q21). For the analyses, the samples were collected at 600 ± 30 m a.s.l. in loam soil with 0% shade. Regarding the presence of branches, 24.54% of (E)-cinnamyl acetate and 20.40% of trans-caryophyllene were identified in sample Q19 compared with 18.03% and 18.89% of the same compounds in the leaves from sample Q18 (without branches). (E)-cinnamyl acetate was the main compound in samples Q20 and Q21 (65% and 12% moisture) with 24.20% and 22.63%, respectively.

**Figure 1:**
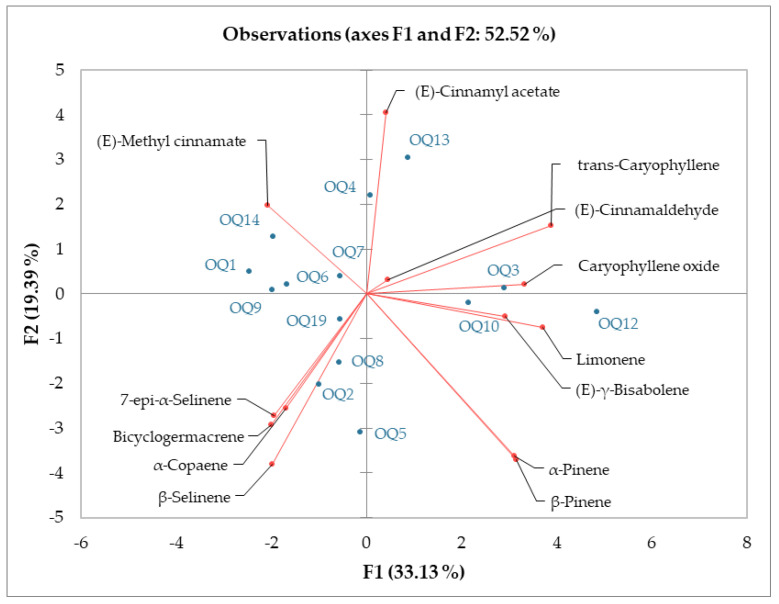
Principal Component Analysis (PCA) of the chemical compositions of the essential oil from ishpingo (*Ocotea quixos*) leaf samples.

The samples of the analysis by type of soil, amount of shade, height, and presence of branches were statistically analyzed employing principal component analysis (PCA) (Figure 1). The K-means clustering analysis and plot established using the first two axes, which accounted for 33.13% and 19.39% of the total variance, suggested the existence of three clusters. Cluster 1 was represented by samples Q1, Q4, Q9, and Q14 in which (E)-methyl cinnamate was one of the main compounds. Cluster 2 was made up of samples Q3 and Q13, which contained trans-caryophyllene and (E)-cinnamyl acetate, and Cluster 3 was made up by the samples that did not have an important representation of these three compounds (15, 18, and 21). The most representative samples in Cluster 3 were Q2, Q5, and Q8 (Figure 1). 

## 3. Discussion

The combination of different concentration percentages of chemical compounds in an essential oil is known as the chemical composition of the essential oil. All samples (Q1–Q21) analyzed in this study showed different chemical compositions. One of the variables that most influences the chemical composition of the EO is type of soil [14]. The concentration percentage of the (E)-methyl cinnamate compound had a statistically significant difference (*p*-value < 0.5) across the samples from different soil types and the percentage of trans-Caryophyllene did not present a significant difference in all samples (*p*-value > 0.5). The percentage of trans-Caryophyllene had a non-significant difference between Q3 (rich clay) and Q4 (sand) samples and between Q1 (poor clay), Q2 (clay), and Q5 (loam) samples. A reduction in the (E)-methyl cinnamate compound and an increase in the trans-Caryophyllene compound were observed when increasing the percentage of clay in the soils. The shade level (shading) exerted a marked influence on the percentage of main compounds 15, 18, and 21 [15]; among all samples (Q11–Q15), a statistically significant difference was determined (*p*-value < 0.5). Differentiating main compounds in essential oil samples of the same species results in the so-called essential oil chemotypes [16]. According to Figure 1, the different environmental conditions produced two chemotypes in the essential oil of the *O. quixos* (E)-methyl cinnamate chemotype and trans-Caryophyllene and (E)-cinnamyl acetate chemotype.

The importance of essential oils lies in their chemical composition [17]; the variety of chemical compounds and concentrations present in the different oils provides unique characteristics to each essential oil. Among the most important characteristics of an essential oil are its biological properties (different biological activities) and its aroma (smell). The aroma is the main characteristic that makes essential oils attractive for use in the cosmetic industry. With reference to this property in the oil from *O. quixus* leaves, the predominance of trans-Cinnamaldehyde (16.62%) gave the essential oil extracted from samples collected in the Amazon province of Pastaza the typical aroma of cinnamon [10], and the predominance of trans-caryophyllene (15.1%) and sabinene (7.6%) conferred a pungent woody aroma to the essential oil of samples collected around the city of Macas in the Amazon province of Morona Santiago [8].

The chemical concentration of essential oils of the species changes due to variations in genetic factors, as well as in ecological and environmental conditions, the most important of these being soil, shade, moisture, seasonal variations, vegetative cycle, temperature, harvest period, and geographical location [18]. The influence of environmental conditions on the chemical composition and of this, in turn, on the biological properties of the essential oil creates the need to study chemical variability depending on environmental variables, in such a way that it is possible to identify the factors responsible for a specific composition. Table 2 shows the great variety of chemical compositions present in the essential oil of ishpingo samples collected under different environmental conditions; the main compound alternated between (E)-cinnamyl acetate, (E)-methyl cinnamate, and trans-caryophyllene depending on the harvesting conditions. According to Sacchetti et al. [8], in the oil from ishpingo leaves collected in the Ecuadorian Amazon, specifically in the city of Macas, the main compounds were trans-caryophyllene with 15.1%, cinnamyl acetate with 11.4%, sabinene with 7.6%, geranial with 5.6%, and trans-Cinnamaldehyde with 5.1%, although the environmental conditions of the species collected were not specified; in comparison with the study by Sacchetti et al., in the present study, geranial, which is an oxygenated terpene, was not detected.

In 2018, Noriega-Rivera et al. [9] evaluated the antioxidant potential of the ishpingo essential oil by the DPPH and ABTS methods; the oil was extracted from fresh leaves collected at the Kutukú biological station in the amazon province of Morona Santiago, and the collection conditions were not specified. The study determined that the *O. quixus* oil has the highest potential of electron scavenging capacity and, according to Noriega et al. [9], this capacity is due to the activity of the trans-caryophyllene (18.22%), α-humulene (16.38%), copaene (4.07), and caryophyllene oxide (3.57) compounds; in relation to this study, in the present research, α-humulene, an ALS compound, was not identified. It should be noted that Noriega et al. did not attribute the antioxidant activity to two of the major compounds also identified: (E)-methyl cinnamate (11.99%) and (E)-cinnamyl acetate (7.00%).

The essential oil from fresh ishpingo leaves collected in the Ecuadorian province of Pastaza used in a concentration of 500 µL/mL reached an average of 94% growth inhibition rate for *Aspergillus oryzae* (ATCC 10124), *Cladosporium cladosporioides* (ATCC 16022), *Fusarium solani* (ATCC 36031), *Rhyzopus stolonifer* (ATCC 6227), *Moniliophthora roreri,* and *Phytophthora* sp.; the last two were isolated from diseased cocoa pods and, in this oil, the main compounds were (E)-cinnamaldehyde (16.62%), trans-methyl isoeugenol (11.94%), trans-caryophyllene (10.59%), α-pinene (9.39%), and β-pinene (6.06%) [10]. The trans-methyl isoeugenol compound (CF: C_11_H_14_O_2_) was not identified in this research.

The larvicidal effect of the *O. quixos* essential oil on *Aedes aegypti* at different concentrations, after 24 h of exposure and expressed as mortality percentage, was reported by Scalvenzi et al.; the mean values obtained were LC_50_ of 75.5 ppm, LC_90_ of 122.56 ppm, and LC_99_ of 181.89 ppm [11]. In this oil obtained from the aerial parts (leaves and twigs) of *O. quixos* collected in the Amazonian region of Pastaza (Ecuador), in unspecified collection conditions, the main compounds were 1,8-cineole (39.2%), sabinene (6.5%), and α-pinene (6.3%). The large amount (approximately 40%) of 1,8-cineole determined by Scalvenzi et al. [11] in this essential oil from samples collected in Pastaza contrasts with the 1.82% (highest percentage, compound 7) of this compound found in the Rich clay samples (Table 2) of the present research. In similar studies, it was determined that eucalyptol (1,8-cineole) (53.49%) may be involved in the larvicidal activity of the *Cinnamomum camphora* essential oil against *Anopheles stephensi* [19] and that the essential oil from *Eucalyptus nitens*, in which 1,8-cineole is present in 22.88%, showed repellent and larvicidal activity against *Aedes aegypti* and *Aedes albopictus* [20]; in these studies, it is suggested that the repellent effect is not only due to the main component, but that it occurs in conjunction with other compounds.

Of the six variables studied, the one that exerted the greatest influence on chemical composition was type of soil [21]. The samples of the five types of soil, namely: Poor clay, Clay, Rich clay, Sand, and Loam, produced essential oils of different chemical compositions with different concentrations for each compound. In most cases, the difference was significant as in the case of the (E)-methyl cinnamate compound. In addition to influencing the individual percentages of each compound, all of the variables (type of soil, height, shade, leaf age, presence of branches, and humidity) also exerted a direct influence on the amount of compound types ALM, ARM, OXM, ALS, ARS, OXS, and OTC (Table 2) [22]. The variable that exerted the least influence on chemical composition was leaf humidity (Table 2) [23].

The influence of environmental, collection, and distillation conditions on the chemical composition in this case determined the presence, or lack thereof, of compounds or groups of compounds [24]. The results of this research revealed that the variables affected the *Ocotea quixus* chemotype and that the EO samples could be divided into two groups (Figure 1): (E)-methyl cinnamate chemotype group that included the sand soil type, 400 m a.s.l. high and 50% of shade samples, and the trans-caryophyllene/(E)-cinnamyl acetate chemotype. 

## 4. Materials and Methods 

### 4.1. Materials

Dichloromethane (DCM) and anhydrous sodium sulfate were purchased from Sigma-Aldrich (San Luis, MO, USA). Helium was purchased from INDURA (Quito, Ecuador). The standard of aliphatic hydrocarbons was purchased from CHEM SERVICE (West Chester, PA, USA) under code M-TPH6X4-1ML (Diesel Range Organics Mixture #2-GRO/DRO). All chemicals were of analytical grade and were used without further purifications.

### 4.2. Soil Type Analysis

Samples of different soil types were analyzed using the Bouyoucos method [25] with the modifications reported by Beretta et al. [26]. The samples were analyzed to determine the size distribution of the mineral particles (texture). The soil samples were dried at 40 °C for 48 h and further ground and sieved to eliminate particles larger than 2 mm in diameter. A blank (containing only water and the dispersing agent) was used to calibrate the hydrometer. The values recorded from the readings were used to calculate the clay, silt, and sand percentages. The calculations were as follows: Sand (%) = 100 − (reading at 40 s × 2 − blank reading) × 100/dried mass of soil; Clay (%) = (reading at 2 h × 2 − blank reading) × 100/dried mass of soil; Silt (%) = 100 − sand (%) − clay (%).

### 4.3. Shade Level Measurements

Shade level was measured using the technique described by Farfán Valencia [27], for which photographs were taken of the treetops in the collection areas. The photographs were then compared with the Visual Shadow Template (PVS, by its acronym in Spanish) [27]. The results were expressed as a shade percentage.

### 4.4. Plant Material

**Figure 2:**
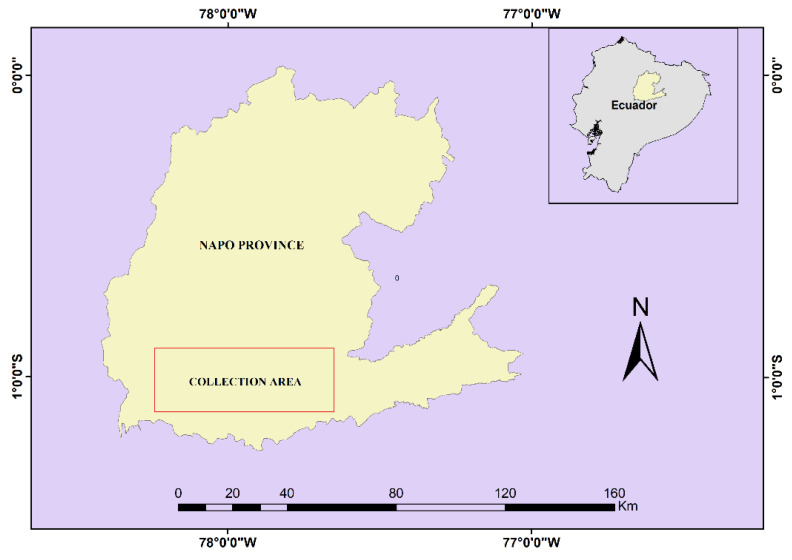
Collection sector of the *Ocotea quixos* leaves in the Ecuadorian Amazon.

The *Ocotea quixos* leaves were collected in six locations distributed in the Talag parish (at a latitude of 1°03′57″ S and a longitude of 77°54′27″ W), the Puerto Misahualli parish (1°01′56″ S, 77°40′13″ W), the Pano parish (1°01′12″ S, 77°51′57″ W), and the Puerto Napo parish (1°02′35″ S, 77°47′38″ W), Tena canton, Napo province (Figure 2) in Ecuadorian Amazon. From each sample, 16 kg were collected and the average environmental conditions of collection were as follows: temperature of 24 °C, pressure of 0.87 atm, annual rainfall of 2700 mm, and relative humidity of 88%. The samples were collected between heights of 370 and 650 m a.s.l. Storage and transfer of the plant material were carried out in airtight plastic containers until they were subjected to postharvest treatment. Transfer and collection temperature was at room temperature (24 °C).

### 4.5. Leaf Age

The leaves were classified into young and old according to their color: those that presented a yellow-green color (RGB values of R: 154, G: 205, B: 50 and CMYK values of C: 0.25, M: 0, Y: 0.76, K: 0.2) were considered as young and those that presented a sea-green color (RGB values of R: 46, G: 139, B: 87 and CMYK values of C: 0.67, M: 0, Y: 0.37, K: 0.45) were considered as old. The term mixture was used for the cases in which the leaves of the entire branch were collected (common collection).

### 4.6. Postharvest Treatments

The postharvest treatments were made immediately after the vegetal material arrived at the laboratory, between 2 and 4 h after being collected, and consisted of the separation of foreign material and degraded leaves. At this stage, the branches were cut to some selected samples. In the cases referring to whole leaves, distillation was carried out on the leaves including the petiole and the branches (twigs) attached to the petiole without the stems. Collection of the leaves together with a part of the twig is the way in which artisan collection of this species is made. The samples that were hydrodistilled at 12% moisture were dried in a drying room at 32 °C until they reached the desired humidity (3–4 days).

### 4.7. Moisture Determination

The moisture of the plant material was determined using the AOAC 930.04-1930 test method: Loss on drying (Moisture) in plants. For moisture determination, an analytical balance (Mettler AC 100, Columbus, OH, USA) was used.

### 4.8. Essential Oil Extraction

For extraction of the essential oil, the vegetal material was hydrodistilled for 340 min in a Clevenger-type device, for which sample mixed with water was boiled to evaporate volatile components, then two layers (aqueous and oil-rich) were obtained and the oil was separated via a separating funnel. Subsequently, the moisture in the essential oil collected was removed by adding anhydrous sodium sulphate and, finally, it was stored in amber-sealed vials at 4 °C to protect it from the light until being used in the subsequent analysis [28]. Three hydrodistillation procedures were carried out for each sample.

### 4.9. Identification of the Chemical Constituents of the Essential Oil

#### 4.9.1. Quantitative Analysis

For the quantitative analysis, an Agilent gas chromatograph (GC) (model 6890N series, Agilent Technologies, Santa Clara, CA, USA) equipped with a flame ionization detector (FID) was used. The GC-FID analyses were performed using a nonpolar Agilent J&W DB-5ms Ultra Inert GC column (30 m, 0.25 mm, 0.25 µm) (5%-phenyl-methylpolyxilosane) and an automatic injector (Agilent 7683 automatic liquid sampler, Agilent Technologies, Santa Clara, CA, USA) in split mode. The samples, 1 µL of solution (1/100, *v*/*v*, EO/DCM), were injected with a split ratio of 1:50. Helium was used as a carrier gas at 1 mL/min in constant flow mode and an average speed of 25 cm/s. The initial oven temperature was maintained at 50 °C for 3 min and then it was heated to 230 °C with a ramp of 3 °C/min, and the temperature was maintained for 3 min until the end. The injector and detector temperatures were 250 °C. Quantification was done by the external standard method using calibration curves generated by running GC analysis of representative compounds.

#### 4.9.2. Qualitative Analysis

For the qualitative analysis, an Agilent gas chromatograph (model 6890N series, Agilent Technologies, Santa Clara, CA, USA) was used, coupled to a mass spectrometer (MS) (quadrupole) detector (model Agilent series 5973 inert, Agilent Technologies, Santa Clara, CA, USA). The GC-MS analyses were performed using a nonpolar Agilent J&W DB-5ms Ultra Inert GC column (30 m, 0.25 mm, 0.25 µm) and an automatic injector (Agilent 7683 automatic liquid sampler, Agilent Technologies, Santa Clara, CA, USA) in split mode. The samples were injected with a split ratio of 1:50. Helium was used as a carrier gas at 0.9 mL/min in constant flow mode and an average speed of 34 cm/s. The operating conditions for the MS were as follows: electron multiplier 1670 eV, 70 eV, mass range 45–350 *m*/*z*, and scan rate 2 scan/s. The MS was provided with a computerized MSD-Chemstation D.01.00 SP1 system. Identification of the oil components was based on a comparison of mass spectrum data with the Wiley 7n libraries from the internal chromatograph database, and mass spectrum data and relative retention indices (RIs) with those of the published literature [29,30,31]. The RI of the compounds was determined based on the homologous of the standard aliphatic hydrocarbons, which were injected after the oils in the same conditions. The RI was obtained through the arithmetic index described by van Den Dool and Dec. Kratz [32] using Equation (1).

(1) $$\mathrm{RI}=100n+100\frac{\left(RTx-RTn\right)}{\left(RTN-RTn\right)}$$

where *n* is the carbon number of the hydrocarbon that elutes before the compound of interest, *RTx* is the retention time (RT) of the compound of interest, *RTn* is the RT of the hydrocarbon that elutes before the compound of interest, and *RTN* is the RT of the hydrocarbon that elutes after the compound of interest.

### 4.10. Statistical Analysis

The procedures of essential oil extraction and identification of chemical constituents were repeated three times. The data were collected in Microsoft Excel, and the measures of central tendency and analysis of variance (ANOVA) were calculated using Minitab 17 (Version 17.1.0., Minitab LLC., State College, PA, USA). All results were expressed as mean values. Principal Component Analysis (PCA) and K-means clustering analysis were performed with the aid of the XLSTAT software (Version 2014.5.03; Addinsoft, Paris, France). PCA was performed with a Pearson matrix and hierarchical ascending classification was conducted with a Euclidian matrix and Ward aggregation.

## 5. Conclusions

The volatile composition of the essential oil from *Ocotea quixus* leaves collected in different environmental conditions was qualitatively and quantitatively determined. It was determined that soil type, amount of shade, height, and leaf age exerted a significant influence on the presence and amount of chemical compounds in the ishpingo essential oil. The variable that exerted the least influence on oil composition was leaf moisture. Based on the results obtained, combinations of the different levels of the variables can be carried out to favor the presence of certain compounds. The present study lays the foundation for the chemotypic classification of the *Ocotea quixos* essential oils. The results of this research demonstrated the need for intensive studies on the influence of the environmental conditions of aromatic species collection on the chemical composition of essential oils.

## Figures and Tables

**Table 1:** Environmental conditions of collection and characteristics of ishpingo (*Ocotea quixos*) leaves of the samples studied.

Sample	Soil Type (Sand-Silt-Clay)	Height (m a.s.l.) ^1^	Shade Level (%)	Leaf Age	Branch Presence	Moisture (%)
OQ1	Poor clay (30-25-45)	600	70	Mixture	With	65 ± 5
OQ2	Clay (25-15-60)	600	70	Mixture	With	65 ± 5
OQ3	Rich clay (10-10-80)	600	70	Mixture	With	65 ± 5
OQ4	Sand (85-10-5)	600	70	Mixture	With	65 ± 5
OQ5	Loam (40-40-20)	600	70	Mixture	With	65 ± 5
OQ6	Loam (40-40-20)	400	0	Mixture	With	65 ± 5
OQ7	Loam (40-40-20)	500	0	Mixture	With	65 ± 5
OQ8	Loam (40-40-20)	600	0	Mixture	With	65 ± 5
OQ9	Rich clay (10-10-80)	400	30	Mixture	With	65 ± 5
OQ10	Rich clay (10-10-80)	600	30	Mixture	With	65 ± 5
OQ11	Loam (40-40-20)	600	0	Mixture	With	65 ± 5
OQ12	Loam (40-40-20)	600	10	Mixture	With	65 ± 5
OQ13	Loam (40-40-20)	600	30	Mixture	With	65 ± 5
OQ14	Loam (40-40-20)	600	50	Mixture	With	65 ± 5
OQ15	Loam (40-40-20)	600	70	Mixture	With	65 ± 5
OQ16	Loam (40-40-20)	600	10	Young	With	65 ± 5
OQ17	Loam (40-40-20)	600	10	Old	With	65 ± 5
OQ18	Loam (40-40-20)	600	10	Mixture	Without	65 ± 5
OQ19	Loam (40-40-20)	600	10	Mixture	With	65 ± 5
OQ20	Loam (40-40-20)	600	10	Mixture	With	65 ± 5
OQ21	Loam (40-40-20)	600	10	Mixture	With	12 ± 2

^1^ Meters above sea level ± 30.

**Table 2:** Chemical composition of the essential oil from ishpingo (*Ocotea quixos*) leaf samples.

CN	Compound	RI	RI^ref^	OQ1	OQ2	OQ3	OQ4	OQ5	OQ6	OQ7	OQ8	OQ9	OQ10	Type
1	α-Pinene	932	932	0.42	2.46	1.99	0.26	3.40	1.39	0.86	1.40	0.80	3.21	ALM
2	Camphene	947	946	-	-	0.13	-	-	-	-	-	-	0.15	ALM
3	Sabinene	968	969	0.15	-	0.33	0.05	-	-	0.20	0.45	0.14	0.14	ALM
4	β-Pinene	973	974	0.55	1.48	1.96	0.34	2.47	0.92	0.96	1.58	1.06	2.73	ALM
5	ρ-Cymene	1019	1020						-	-	-	0.26	-	ARM
6	Limonene	1023	1024	0.32	0.49	1.20	0.74	0.47	0.31	0.30	0.44	0.36	0.55	ALM
7	1,8-Cineole	1025	1026	1.54	0.60	1.82	0.63	-	1.13	1.03	1.29	1.05	1.29	OXM
8	(E)-β-Ocimene	1043	1044	-	-	0.16	-	-	0.16	-	-	-	-	ALM
9	Linalool	1093	1095	0.29	-	0.13	-	-		0.56	0.71	-	0.14	OXM
10	Terpinen-4-ol	1172	1174	0.26	-	0.23	-	-	0.24	-	-	0.25	0.29	OXM
11	α-Terpineol	1189	1186	0.46	0.27	0.54	0.20	-	0.40	0.23	-	0.44	0.55	OXM
12	(E)-Cinnamaldehyde	1268	1267	8.19	9.00	12.68	3.61	1.33	1.97	3.62	2.14	4.02	7.52	OTC
13	α-Cubebene	1345	1345	0.22	0.35	0.77	-	-	-	-	-	0.44	0.21	ALS
14	α-Copaene	1372	1374	6.76	3.32	3.44	1.84	6.30	0.31	1.52	4.13	1.39	0.84	ALS
15	(E)-Methyl cinnamate	1376	1376	29.79	16.00	12.08	37.25	6.16	7.32	6.49	6.57	37.91	8.85	OXM
16	(Z)-Cinnamyl acetate	1388	1388	-	0.93	-	-	0.85	1.31	0.70	0.72	-	0.19	OTC
17	α-cis-Bergamotene	1410	1411	-	-	0.15	-	1.92	-	-	0.81	-	-	ALS
18	trans-Caryophyllene	1415	1417	12.16	14.14	21.19	21.16	13.41	12.71	20.22	17.55	8.77	21.61	ALS
19	α-Guaiene	1435	1437	-	-	0.17	-	3.23	-	-	1.66	-	-	ALS
20	6,9-Guaiadiene	1442	1442	1.65	9.64	2.05	2.95	5.33	18.78	2.49	-	-	0.89	ALS
21	(E)-Cinnamyl acetate	1445	1443	12.14	9.95	15.31	11.60	5.96	26.68	24.20	11.86	13.33	23.53	OTC
22	(E)-β-Farnesene	1458	1454	-	0.41	0.63	-	-	-	0.47	-	0.44	0.54	ALS
23	trans-Cadina-1(6),4-diene	1475	1475	-	-	-	0.27	0.95	2.16	0.63	-	-	0.35	ALS
24	β-Chamigrene	1478	1476	0.44	-	-	-	0.79	0.19	0.75	0.63	-	0.21	ALS
25	β-Selinene	1489	1489	3.14	13.24	1.03	1.76	9.37	10.15	7.93	8.95	13.16	5.28	ALS
26	Viridiflorene (=Ledene)	1492	1496	-	0.46	0.39	0.34	2.91	0.41	0.5	1.8	0.37	0.43	ALS
27	Bicyclogermacrene	1496	1500	2.19	6.88	3.54	3.33	3.89	6.52	4.79	5.13	4.48	3.65	ALS
28	Anisyl propanoate	1510	1511	5.12	0.27	2.77	0.25	-	-	9.89	9.53	1.47	5.10	OTC
29	δ-Amorphene	1511	1511	-	0.45	1.19	0.31	1.00	0.28	-	-	1.07	0.53	ALS
30	(Z-)-γ-Bisabolene	1515	1514	1.15	0.58	0.20	0.35	-	0.76	-	2.14	-	0.76	ALS
31	7-epi-α-Selinene	1520	1520	5.00	1.75	0.61	0.41	5.38	0.32	3.03	5.03	0.43	0.57	ALS
32	δ-Cadinene	1521	1522	-	-	0.40	0.21	-	-	-	-	0.29	-	ALS
33	(E)-γ-Bisabolene	1527	1529	0.96	3.05	5.30	0.38	1.40	0.42	2.93	2.66	1.75	2.54	ALS
34	α-Calacorene	1540	1544	1.18	0.32	-	0.08	5.01	-	0.45	3.02	-	-	ARS
35	Selina-3,7(11)-diene	1543	1545	0.27	0.34	0.53	0.47	-	0.20	-	-	0.44	0.37	ALS
36	Elemol	1549	1548	-	0.25	-	0.39	-	0.70	-	-	-	0.22	OXS
37	Pentyl salicylate	1570	1574	-	-	0.46	0.27	-	-	-	-	0.39	0.44	OTC
38	Spathulenol	1575	1577	0.31	-	0.51	1.34	2.87	0.29	0.70	2.15	0.72	0.21	OXS
39	Caryophyllene oxide	1580	1582	1.49	1.37	4.76	6.94	4.63	1.29	2.05	3.60	3.01	4.15	OXS
40	Thujopsan-2-β-ol	1586	1588	-	-	0.82	0.71	1.51	-	-	0.97	0.29	0.15	OXS
41	Humulene epoxide II	1605	1608	-	0.70	-	0.37	1.72	0.90	-	0.56	-	0.12	OXS
42	α-Corocalene	1620	1622	0.88	-	-	-	0.81	-	0.27	0.68	-	-	ARS
43	Muurola-4,10(14)-dien-1β-ol	1626	1630	-	-	-	0.11	-	-	-	-	-	-	OXS
44	Exalatacin	1652	1655	-	-	-	-	0.81	-	0.33	-	-	-	OTC
45	trans-Calamenen-10-ol	1664	1669	-	-	-	-	1.62	-	-	-	-	-	OXS
46	Cadalene	1671	1675	-	-	-	-	1.66	-	-	0.39	-	-	ARS
47	Benzyl benzoate	1755	1759	0.35	-	-	-	-	-	-	-	-	0.71	OTC
Aliphatic monoterpene hydrocarbons (ALM)				1.45	4.44	5.76	1.39	6.34	2.78	2.31	3.87	2.36	6.79	
Aromatic monoterpene hydrocarbons (ARM)				-	-	-	-	-	-	-	-	0.26	-	
Oxygenated monoterpenes (OXM)				32.34	16.87	14.81	38.08	6.16	9.09	8.31	8.56	39.66	11.12	
Aliphatic sesquiterpene hydrocarbons (ALS)				33.94	54.61	41.58	33.78	55.89	53.22	45.26	50.48	33.03	38.78	
Aromatic sesquiterpene hydrocarbons (ARS)				2.06	0.32	-	0.08	7.48	-	0.72	3.70	-	-	
Oxygenated sesquiterpene (OXS)				1.80	2.31	6.090	9.85	12.35	3.18	2.75	7.66	4.02	4.86	
Other compounds (OTC)				25.8	20.16	31.22	15.72	8.94	29.95	38.73	24.25	19.23	37.49	
Total identified				97.39	98.70	99.47	98.92	97.17	98.22	98.08	98.53	98.55	99.03	
**CN**	**Compound**		**OQ11**	**OQ12**	**OQ13**	**OQ14**	**OQ15**	**OQ16**	**OQ17**	**OQ18**	**OQ19**	**OQ20**	**OQ21**	**Type**
1	α-Pinene		1.40	4.07	0.94	0.34	3.40	4.51	5.76	0.60	1.13	0.86	0.76	ALM
2	Camphene		-	0.22	-	-	-	0.24	0.28	-	-	-	-	ALM
3	Sabinene		0.45	0.14	-	0.12	-	0.23	0.23	0.06	0.32	0.20	0.30	ALM
4	β-Pinene		1.58	2.62	0.79	0.50	2.47	2.8	3.30	0.71	1.27	0.96	0.75	ALM
5	ρ-Cymene		-	0.20	-	-	-	0.20	-	-	-	-	-	ARM
6	Limonene		0.44	1.36	0.18	0.29	0.47	0.56	1.96	0.33	0.37	0.30	0.32	ALM
7	1,8-Cineole		1.29	1.29	0.75	0.74	-	1.16	1.73	0.93	1.16	1.03	0.68	OXM
8	(E)-β-Ocimene		-	-	-	-	-	-	-	0.13	-	-	-	ALM
9	Linalool		0.71	0.07	-	-	-	0.10	-	0.17	0.64	0.56	0.66	OXM
10	Terpinen-4-ol		-	-	-	0.15	-	-	-	0.23	-	-	-	OXM
11	α-Terpineol		-	0.37	0.28	0.29	-	0.33	0.34	0.29	0.11	0.23	0.22	OXM
12	(E)-Cinnamaldehyde		2.14	3.51	2.25	8.97	1.33	2.21	2.18	5.05	2.88	3.62	2.68	OTC
13	α-Cubebene		-	0.54	0.20	1.06	-	0.54	0.45	0.53	-	-	0.22	ALS
14	α-Copaene		4.13	0.83	0.56	4.48	6.30	0.77	1.53	3.47	2.82	1.52	2.62	ALS
15	(E)-Methyl cinnamate		6.57	1.82	7.84	17.05	6.16	0.38	2.15	9.48	6.53	6.49	6.31	OXM
16	(Z)-Cinnamyl acetate		0.72	0.23	-	-	0.85	-	0.48	0.31	0.71	0.70	0.56	OTC
17	α-cis-Bergamotene		0.81	0.25	0.33	-	1.92	0.26	0.24	-	-	-	-	ALS
18	trans-Caryophyllene		17.55	30.07	21.44	16.62	13.41	37.02	26.63	20.4	18.89	20.22	20.48	ALS
19	α-Guaiene		1.66	0.24	-	0.23	3.23	0.24	0.28	0.12	0.83			ALS
20	6,9-Guaiadiene		-	2.45	2.13	1.84	5.33	3.68	-	2.4	1.25	2.49	2.47	ALS
21	(E)-Cinnamyl acetate		11.86	18.21	41.65	28.62	5.96	14.27	8.68	24.54	18.03	24.2	22.63	OTC
22	(E)-β-Farnesene		-	0.91	0.79	-	-	1.05	0.37	0.17	0.23	0.47	-	ALS
23	trans-Cadina-1(6),4-diene		-	0.44	1.71	-	0.95	0.57	-	0.24	0.61	0.63	0.60	ALS
24	β-Chamigrene		0.63	-	-	0.38	0.79	-	-	0.70	0.69	0.75	1.00	ALS
25	β-Selinene		8.95	4.40	1.74	3.68	9.37	2.46	9.70	6.87	8.44	7.93	9.71	ALS
26	Viridiflorene (=Ledene)		1.80	-	-	0.65	2.91	0	-	0.73	1.15	0.50	0.59	ALS
27	Bicyclogermacrene		5.13	2.30	1.47	4.34	3.89	1.59	3.74	5.20	4.96	4.79	5.95	ALS
28	Anisyl propanoate		9.53	9.03	3.53	1.46	-	8.35	9.22	5.55	9.71	9.89	8.49	OTC
29	δ-Amorphene		-	1.53	1.40	-	1.00	1.54	1.75	-	-	-	-	ALS
30	(Z-)-γ-Bisabolene		2.14	-	-	0.93	-	-	-	1.44	1.94	-	1.70	ALS
31	7-epi-α-Selinene		5.03	0.54	0.83	3.39	5.38	0.58	0.63	3.71	4.03	3.03	3.84	ALS
32	δ-Cadinene			0.08		0.47		-	0.30	0.23	-	-	-	ALS
33	(E)-γ-Bisabolene		2.66	3.42	3.68	0.77	1.40	3.40	3.82	1.69	2.79	2.93	1.24	ALS
34	α-Calacorene		3.02	-	-	0.38	5.01	0	-	0.47	1.73	0.45	0.45	ARS
35	Selina-3,7(11)-diene		-	0.49	0.39	-	-	0.56	0.63	-	-	-	-	ALS
36	Elemol		-	0.20	-	-	-	0.13	-	-	-	-	-	OXS
37	Pentyl salicylate		-	0.21	0.17	-	-	0.32	0.69	-	-	-	-	OTC
38	Spathulenol		2.15	0.31	0.23	-	2.87	0.32	0.49	0.37	1.42	0.70	0.70	OXS
39	Caryophyllene oxide		3.60	6.63	3.29	0.99	4.63	8.23	10.32	1.31	2.82	2.05	1.45	OXS
40	Thujopsan-2-β-ol		0.97	0.37	-	0.23	1.51	0.55	0.31	0.11	0.48	-	0.30	OXS
41	Humulene epoxide II		0.56	0.25	-	-	1.72	0.37	0.39	-	0.28	-	-	OXS
42	α-Corocalene		0.68	-	-	0.39	0.81	-	-	0.34	0.47	0.27	0.36	ARS
43	Muurola-4,10(14)-dien-1β-ol		-	-	-	-	-	-	-	-	-	-	-	OXS
44	Exalatacin		-	-	-	-	0.81	-	-	0.32	0.16	0.33	0.58	OTC
45	trans-Calamenen-10-ol		0.39	-	-	-	1.62	-	-	-	0.20	-	-	OXS
46	Cadalene		-	-	-	-	1.66	-	-	-	-	-	-	ARS
47	Benzyl benzoate		-	-	0.23	-	-	-	-	0.37	-	-	0.78	OTC
Aliphatic monoterpene hydrocarbons (ALM)			3.87	8.41	1.92	1.25	6.34	8.34	11.53	1.83	3.09	2.31	2.13	
Aromatic monoterpene hydrocarbons (ARM)			-	0.20	-	-	-	0.20	-	-	-	-	-	
Oxygenated monoterpenes (OXM)			8.56	3.54	8.87	18.23	6.16	1.97	4.22	11.09	8.44	8.31	7.87	
Aliphatic sesquiterpene hydrocarbons (ALS)			50.48	48.48	36.67	38.82	55.89	54.27	50.06	47.89	48.62	45.26	50.42	
Aromatic sesquiterpene hydrocarbons (ARS)			3.70	-	-	0.78	7.48	-	-	0.81	2.21	0.72	0.81	
Oxygenated sesquiterpene (OXS)			7.66	7.76	3.52	1.22	12.35	9.60	11.51	1.79	5.21	2.75	2.45	
Other compounds (OTC)			24.25	31.19	47.83	39.04	8.94	25.15	21.24	36.13	31.49	38.73	35.72	
Total identified			98.53	99.6	98.81	99.34	97.17	99.53	98.56	99.54	99.06	98.08	99.39	

CN: Compound Number; RI: Calculated Retention Indices; RI^ref^: References Retention Indices; -: Not detected.

## Data Availability

Data is available from the authors upon reasonable request.

## Sample Availability

Samples of the *Ocotea quixus* essential oil are available from the authors.
